# Effects of environmental regulation on adjustment of agricultural industrial structure——An empirical study based on panel data of 31 provinces in China

**DOI:** 10.1371/journal.pone.0296189

**Published:** 2024-04-18

**Authors:** HongYe Tan, Xin Jing

**Affiliations:** 1 School of Business Administration, Shandong Technology and Business University, Yantai, China; 2 School of Public Administration, Shandong Normal University, Jinan, China; Qufu Normal University, CHINA

## Abstract

Using the panel data of 31 provinces in China between 2000 and 2018, this study theoretically and empirically analyses the impact of environmental regulations on the adjustment of the agriculture industrial structure from the perspectives of rationalisation and optimisation. Overall, variability in the impact of environmental regulation on the adjustment of the agricultural industrial structure is identified: a negative influence on the rationalisation of agricultural industrial structure and a positive influence on the optimisation of agricultural industrial structure. The impact of environmental regulations on the adjustment of agricultural industrial structure also reflects "large country characteristics": environmental regulations are more significant at the medium and low industrial structure levels. Environmental regulation significantly impacts the rationalisation of agricultural industrial structure in central and western regions and its optimisation in eastern and central regions. Through the panel threshold model test, this paper further finds that the effect of environmental regulations on the adjustment of agricultural industrial structure is not invariant. However, there is a non-linear relationship with significant threshold characteristics. Based on the above results, some countermeasures are suggested.

## Introduction

Agricultural industrial structure adjustment conforms to the laws of development and basic evolutionary logic. It is an inevitable choice for countries at specific economic and social development stages. As the central contradiction of China’s agriculture has changed from low output to structural imbalance, adjusting the industrial structure has become imperative. The imbalances in the proportion structure of agricultural input factors, the coordinated development structure of the industrial chain, the structure of the relationship between the government and the market, and the macro-control mechanism have become more and more evident with the economic and social development in China [1]. The adjustment and upgrading of the existing agricultural structure lags behind the pace of upgrading the consumption structure, and the international competitiveness of agricultural products is insufficient. The unsustainable ecological problems of agricultural production have intensified, the construction of agricultural property rights and factor markets is lagging, and other agricultural supply-side structural problems are prominent [2]. Hence, the agricultural structure has entered an inevitable stage where it has to be adjusted and upgraded.

However, in promoting the adjustment of the agriculture industry’s 653 structures and activating the flow of resources and factors, the problems of agricultural resources and ecological environment protection have become more prominent, such as the degradation of cultivated land quality, shortages of agricultural water, severe agricultural non-point source pollution, and fragile ecological environments. High-quality agricultural development requires correctly handling the relationship between resources and environmental protection and coordinating the development of industrial structure adjustment. The report of the 20th National Congress of the Communist Party of China requires the industry to ’promote the construction of a beautiful China, coordinate industrial restructuring, pollution control and ecological protection, and promote ecological priority, conservation, and intensive green and low-carbon development’. It is inherently inevitable and necessary to support the structural adjustment of agricultural industries and the high-quality development of agriculture with the concept of green development. Correspondingly, China has successively issued a series of environmental regulation policies and implemented environmental regulation measures such as ’the battle of agricultural non-point source pollution control’ and ’the action of livestock manure resource utilisation. At the same time, the ’No. 1 Central Document’ issued for 2022 also re-emphasises ’promoting the green development of agriculture and rural areas and strengthening the comprehensive management of agricultural non-point source pollution’. It can be seen that promoting the green development of agriculture and realising the sustainable utilisation of agricultural resources have become essential for national industrial policy.

Therefore, in adjusting the structure of agriculture, the negative externalities of environmental pollution cannot be entirely solved by market mechanisms. In other words, agricultural environmental protection and pollution control cannot be achieved without government assistance. In this context, it is necessary to explore the relationship between environmental regulation and the industrial structure adjustment of agriculture. Based on macro panel data, this paper empirically analyses the impact of environmental regulation on the adjustment of the industrial structure, heterogeneity characteristics, and threshold effects to provide theoretical and policy support for optimising and upgrading the agriculture industry.

## Literature review

Environmental regulation is a way for the government to solve the negative externalities arising from economic subjects’ behaviour, improve resource allocation efficiency, and achieve the twin goals of environmental improvement and economic development through administrative orders and standard formulation [3]. The economic and social impacts of environmental regulation have been extensively studied. One such research area is the relationship between environmental regulation and corporate behaviour. Most scholars have focused on the impact of environmental regulations on internal investment, enterprise factor allocation, enterprise technological innovation, total factor productivity, regional location regulation, and other behaviours after the internalisation of external costs [4, 5]. The second area of research is the economic effect of environmental regulation. Some studies confirmed that environmental regulation can improve the competitiveness of enterprises and promote macroeconomic development [6, 7]. However, other scholars believe that environmental regulation has a limited impact on economic development and can even have an inhibitory effect [4, 8]. The third area of research is the social effects of environmental regulation. Environmental regulations affect not only corporate behaviour and economic development but also the corresponding social impacts of regulations, including pollution control, carbon emissions, green development, income consumption, residents’ employment, and population behaviour [9, 10]. Therefore, the evaluation of environmental regulation policies must have a sociological perspective.

Regarding the relationship between environmental regulations and industrial structure adjustments, Walter and Ugelow proposed the ’pollution paradise hypothesis’, which argues that environmental regulation will lead to higher production costs for enterprises, causing the inevitable industrial transfer of many heavily polluting enterprises [11]. In contrast, Michael Porter posited the ’Porter hypothesis’, which argues that strict and appropriate environmental regulation can stimulate innovation, offset environmental regulations’ costs, promote technological innovation and production efficiency, and lead to industrial optimisation and upgrading [12]. Therefore, scholars have conducted in-depth research on the industrial structure adjustment effect of environmental regulation from different perspectives and using various data, achieving some beneficial results [13–15]. In particular, many scholars have used Chinese data to empirically analyse how environmental regulations impact and act on industrial production, industrial transfer, industrial structure optimisation, and other aspects [16, 17].

Agricultural industrial structure is considered essential to environmental pollution due to the use of fertilisers and other chemicals [18], energy conservation and income growth [19]. Spatial econometrics analysis shows that adjusting agricultural industrial agglomeration can upgrade energy efficiency while reducing carbon emissions [20, 21], contributing to China’s sustainable agricultural development. The studies above show the significance of the Chinese government taking measures to readjust the agricultural, industrial structure [22]. Environmental regulation is appropriate for the abovementioned features. However, few studies have directly analysed the impact of environmental regulations on the adjustment of agricultural industrial structure. Existing studies have focused heavily on analysing the effects of environmental regulations on resource use of agricultural waste, surface pollution, agricultural technology, and industrial efficiency, for example [23–25]. Only a few scholars have empirically analysed the spatial layout and industrial development of environmental regulation, taking the industries of hogs, livestock, and aquaculture as examples [26–28]. Similarly, Yu and Qi found that the project of returning farmland to forestry can expand the grain-to-economy ratio in the agricultural industry structure, with specific crowding-out and spillover effects [29]. As for the impact of environmental regulation on the linkage and combination structure of different agricultural industries, there is a lack of in-depth research and empirical analysis.

In summary, regarding environmental regulation, scholars focus on the connotation, type and strategy choice of environmental regulation, the economic and social impact of environmental regulation, the decision-making behaviour of enterprises, and the upgrading of industrial structures. Concerning agricultural structure, scholars focus on the necessity, connotation, influencing factors and functions of agricultural structure adjustment. Highlighting the relationship between environmental regulation and agricultural industrial structure, scholars mainly analysed the structural adjustment of the hog industry and the impact of environmental regulation on agricultural green production behaviour and agricultural development. Most studies adopt empirical tools such as panel data regression or spatial econometric models to analyse causality, intermediary effect and spillover effect based on macro data. However, the following defects and deficiencies still exist in current theories and empirical studies: First, few studies directly explore the impacts of environmental regulations on agricultural industrial restructuring. Secondly, few studies explore different impacts of environmental regulations on agricultural industrial restructuring from the perspective of the degree of environmental regulations and regional heterogeneity. Finally, few studies involve empirical tests based on China’s macro long-panel data, considering potential endogenous problems.

The marginal contribution and potentials of this study are multiple. By distinguishing macro-industrial structure from industrial transformation, this study highlights the impact of environmental regulations on the adjustment of agricultural industrial structure, which consists of agriculture, forestry, livestock, and fisheries. Additionally, distinct from a linear correlation study, this analysis empirically examines environmental regulations’ heterogeneous and non-linear effects on agricultural industrial restructuring based on long-panel data. Finally, corresponding policy recommendations are proposed to compensate for the lack of policy research on structural adjustment in the agriculture industry.

## Theoretical analysis

The agricultural industrial structure reflects the composition and correlation of different production departments within the industry, including agriculture, forestry, animal husbandry and side-line fishery in a broad sense and agricultural products in a narrow sense. Adjustment of the agricultural industrial structure refers to the continuous optimisation and coordination of resources and elements among various agricultural departments to optimise industrial development.

Based on the "Allotey-Clark theorem", "Kuznets’ law", "industrial structure evolution theory", "institutional change theory", and other related theories, the adjustment of agricultural industrial structure follows the fundamental law of industrial evolution. With the rapid transfer of labour factors to the secondary and tertiary industries driven by comparative advantage, the allocation of factors within the agricultural industry changes accordingly. Additionally, with changes in macro market demand and national agricultural development strategy goals, the agricultural industrial structure naturally forms the characteristics of a dynamic evolution process. As the adjustment of agricultural industrial structure arises from internal factors such as resource endowment, infrastructure, agricultural technology and capital input, it also cannot be separated from the external conditions of industrial policy, market environment, and economic development, and is the inevitable result of the comprehensive influence of multiple variables. Of course, the main body of agricultural industrial structure adjustment is agricultural producers, and it is also influenced by external forces such as the government and the public. As an essential part of agricultural systems and industrial policies, environmental regulations will inevitably have a specific impact on the adjustment of agricultural industrial structure. From the perspective of industrial economics, the adjustment of industrial structure reflects the process of leading the evolution and replacement of industrial sectors and guiding the coordinated development of various industries, which primarily involves the two adjustment directions: industrial structure rationalisation and industrial structure upgrading [30]. Previous studies also mainly explore the evolution trend of industrial structure around the rationalisation and upgrading dimensions. The current study focuses on the impact of environmental regulation on the rationalisation and upgrading of agricultural industrial structure.

### (1) Environmental regulation and rationalisation of agricultural industrial structure

Rationalisation of agricultural industrial structure refers to the free flow and rational allocation of production factors and input resources among different production departments. It is a dynamic process that guides industrial linkages from uncoordinated to reasonable combinations. The rationalisation of industrial structure emphasises the rational allocation of production factors and the coordinated development of various industries, which requires improving the coupling degree of input and output structure of factors. On the one hand, according to “compliance cost theory” [31] with the increase of environmental regulation intensity, the input cost of agricultural industry factors increases while the income decreases. Due to the regional restrictions and weak qualitative characteristics of the agricultural industry, it is difficult for the agricultural industry to reduce regulatory costs by transferring pollution to other areas or sectors. The regulatory investment can only be increased to maintain income, resulting in a ’crowding out effect’ [32] that will inhibit the free flow and reasonably allocate factors. The effect leads to an imbalance in the existing factor allocation structure of the agricultural industry. In contrast, implementing environmental regulation has reconstructed the input structure of various agricultural industries to some extent, guiding the green development of the industry. However, the green output structure struggles to adjust and achieve economic benefits quickly, so the input structure and output structure cannot be fully coupled. The implementation of environmental regulation makes the limited production factors of the economy, and society redistributed within the agricultural industry, breaking the original factor allocation and output structure, which is not conducive to rationalising agricultural industrial structure in the short term. Consequently, Hypothesis 1 is that environmental regulations break the existing structural configuration among agricultural industries and negatively impact the rationalisation of agricultural industrial structure.

### (2) Environmental regulation and optimisation of agricultural industrial structure

The optimisation of agricultural industrial structure means that the agricultural industry is no longer limited to planting, breeding or agricultural production. Through technological innovation and factor upgrading, the agricultural industry is adapting with modernisation, in the industrialisation direction of high intensification, high technology, high processing, and high added value [33]. It can be seen from the ’innovation compensation effect’ that appropriate environmental regulation intensity can induce technological innovation behaviours, compensating for the regulation costs generated by environmental regulations through innovation compensation effects, thereby enhancing the competitiveness of enterprises (Wagner, 2007 [34]). Similarly, implementing environmental regulation can guide agricultural machinery, green technology, digital agriculture, intelligent management, and other advanced elements to be invested in the agricultural industry [35]. With intensive labour and resources, traditional agriculture is gradually eliminated, agricultural production efficiency is further improved, and the agricultural industry has a noticeable trend towards upgrading and modernisation. The knowledge and technology spillovers generated by agricultural innovation will, in turn, demonstrate and guide the transformation of the production methods of surrounding farmers. Factors and industrial upgrading will further exert spillover effects in a larger space, once again optimising and upgrading the agricultural industry. Implementing environmental regulation will produce a “consumption substitution effect” [36], which guides consumers’ consumption concept and structure to environmentally friendly agricultural products on the demand side of the market. To meet the needs of consumers for green consumption, the agricultural industrial structure will evolve to produce all kinds of green, high-quality, high value-added high-quality agricultural products. Hypothesis 2 is that environmental regulations can exert the innovation compensation effect and knowledge spillover effect, which, combined with green consumption transformation, will optimise agricultural industrial structure.

### (3) Non-linear impact of environmental regulation

Previous studies have found that environmental regulation and industrial structure adjustment are not a fixed linear relationship, but rather exhibit characteristics such as non-linearity and spatial spillover [5]. Similarly, the impact of environmental regulations on the structure of the agricultural industry is due to the strengthening of environmental regulations leading to an increase in the prices of agricultural production factors, resulting in an adjustment in agricultural production behaviour. Therefore, with the continuous introduction of environmental regulation policies and increasing the intensity of environmental regulation, the impact of environmental regulation on the adjustment of agricultural industrial structure may also exhibit non-linear characteristics. In other words, the linear relationship does not truly reflect the long-term impact of environmental regulations on the adjustment of agricultural industrial structure. The ’compliance cost effect’ or ’innovation compensation effect’ will change with the continuation of environmental regulation behaviour, which manifests as a non-linear impact on the adjustment of agricultural industrial structure.

Based on this, a third hypothesis is proposed that as the intensity of environmental regulation changes, environmental regulation has a non-linear impact on the rationalisation and optimisation of agricultural industrial structure.

## Material and methods

### Variable setting

#### (1) Dependent variable

The Theil index is used to measure the rationalisation of the agriculture industrial structure (TL) [Eq (1)]. TL = 0 means that the labour production ratio of each subdivided industry is consistent with the agricultural output ratio, the industrial coordination is the highest, and the industrial structure is the most reasonable. Conversely, agricultural industrial structure tends to be unreasonable. Due to the lack of data on the number of labour forces in various agricultural industries, this study used relevant research for reference [37], replacing the intermediate consumption with the value-added ratio:

$$TL=\sum_{i=1}^{n}\frac{Y_{i}}{Y}ln(\frac{Y_{i}}{Y}/\frac{L_{i}}{L})$$ (1)

where Y_i_ denotes the output value of agricultural subdivided industries, Y denotes the gross value of agricultural output, L_i_ denotes the number of labour forces in agricultural-subdivided industries, and L denotes the total labour force in the agricultural industry. Due to the lack of macro statistical data on the number of labour forces in agricultural-subdivided industries, a ratio of agricultural added value to intermediate consumption was used to measure labour productivity.

The optimisation of agricultural industrial structure (EI) refers to the degree to which the agricultural industry tends to develop sub-industries with high added and output values. Previous studies primarily used the ratio of grain to crops, the ratio of the output value of agriculture and animal husbandry, and the ratio of agricultural product processing and agricultural output value to represent agricultural industrial structure upgrading [38, 39]. Considering the validity of indicators and the availability of data, based on the practice of Cao Fei et al. [40], this study adopts the proportion of the output value of farming, forestry, animal husbandry and fishery service industry in the total agricultural output value as the proxy index for agricultural industrial structure upgrading.

#### (2) Explanatory variable

Previous reports primarily used indicators such as the number of environmental regulation policies, the investment amount of agricultural environmental governance, the return rate of rural water transformation, the amount of fertiliser applied, and the content of excess nitrogen in cultivated land to measure the environmental regulation intensity. Considering the indicators’ validity and data availability, this study uses agricultural carbon emissions as a proxy variable for environmental regulation. Such a proxy variable is rational for several reasons. Environmental regulation and carbon emissions are closely related, with a significant negative correlation [41, 42]. In contrast, many scholars directly take pollution emission or production intensity as the measurement index of environmental regulation in their empirical studies [43]. Therefore, utilising the protocols of Zhang Jinxin and Wang Hongling [44], the current study adopts a calculation method recommended by the *2006 IPCC Guidelines for National Greenhouse Gas Inventories*. The total amount of agricultural carbon emission, calculated by multiplying carbon emission sources and their carbon emission coefficient in agricultural production, represents environmental regulation intensity (ER). The carbon emission coefficients of different carbon emission sources are shown in Table 1.

**Table 1:** Agricultural carbon emission sources and related emission coefficients.

Carbon emission source	Carbon emission factor	Reference source
Fertiliser	0.8956kg/kg	Oak Ridge National Laboratory
Pesticide	4.9341 kg/kg	Oak Ridge National Laboratory
Agricultural film	5.1800 kg/kg	Institute of Agricultural Resources and Ecological Environment, Nanjing Agricultural University
Agricultural diesel	0.5927 kg/kg	IPCC(200)
Agricultural sown area	3.1260 kg/hm^2^	School of Biology and Technology, China Agricultural University
Agricultural irrigation area	25.000 kg/ hm^2^	Dubey (2009) [45]

#### (3) Control variable

In existing research, national income level, government policy intervention degree, resource endowment, agricultural modernisation, and other factors influence industrial structure [46, 47]. This paper introduces a series of control variables: urbanisation (URB), represented by the proportion of non-agricultural population in the total population; agriculture subsidy (SUB), represented by financial investment to farming, forestry, animal husbandry and fishery; human capital (CAP), represented by average years of education in rural areas; level of regional economic development (ECO), represented by regional GDP per capital; resource endowment (RES), represented by agriculture machine value taken by rural per capita.

## Model construction

A panel regression model based on macro data was established to examine the linear relationship between environmental regulation and agricultural industrial structure adjustment from the two dimensions of rationalisation and optimisation:

$$TL_{it}=\beta_{0}+\beta_{1}{ER}_{it}+\sum \beta_{n}X_{it}+\mu_{i}+e_{t}+\varepsilon_{it}$$ (2)

$$EI_{it}=\beta_{0}+\beta_{1}{ER}_{it}+\sum \beta_{n}X_{it}+\mu_{i}+e_{t}+\varepsilon_{it}$$ (3)

where TL_it_ denotes the rationalisation of agricultural industrial structure; EI_it_ denotes the optimisation of agricultural industrial structure; ER_it_ denotes environmental regulation intensity; X_it_ denotes control variables at the provincial level; μ_i_ denotes individual fixed effects; e_t_ denotes time fixed effects; and ε_it_ denotes the error term.

In addition to the linear relationship, many scholars have studied and discovered the non-linear effects of environmental regulation. They believe that with changes in the intensity of environmental regulation, there would be specific structural changes in its role. If so, would the impact of environmental regulation on the adjustment of agricultural industrial structure also have non-linear characteristics? Would there be differences in the impact intensity of environmental regulation within different regulatory intensities? Therefore, based on benchmark panel regression, a non-dynamic panel threshold regression model was developed based on the Hansen model to test the non-linear relationship between environmental regulation and agricultural industrial structure adjustment:

$$TL_{it}=\beta_{0}+\beta_{1}{ER}_{it}∙I(q_{it}\leq \gamma )+\beta_{2}{ER}_{it}∙I(q_{it}>\gamma )+\sum \beta_{n}X_{it}+\mu_{i}+e_{t}+\varepsilon_{it}$$ (4)

$$EI_{it}=\beta_{0}+\beta_{1}{ER}_{it}∙I(q_{it}\leq \gamma )+\beta_{2}{ER}_{it}∙I(q_{it}>\gamma )+\sum \beta_{n}X_{it}+\mu_{i}+e_{t}+\varepsilon_{it}$$ (5)

where I(•) denotes indicator function; q_it_ denotes threshold variable; γ denotes threshold to be estimated.

### Data source

The data in this study were sourced from the ’China Statistical Yearbook’ and ’China Rural Statistical Yearbook’ are limited by data availability, the panel data of 31 Chinese provinces from 2000 to 2018, excluding Hong Kong, Macau, and Taiwan, were used. An empirical analysis using Stata16 software was undertaken to test the linear and non-linear relationship between environmental regulation and agricultural industrial structure adjustment. Table 2 shows the data descriptions of all variables.

**Table 2:** Main variable descriptions.

Variable	Description	Average value	Standard deviation
TL	Agricultural structural rationalisation	1.33	0.32
EI	Agricultural structural optimisation	0.03	0.02
ER	Environmental regulatory intensity	5.10	1.14
URB	Urbanisation: expressed by the proportion of non-agricultural population in the total population in each province	0.49	0.15
SUP	Agricultural support intensity: expressed by financial investment quota of agriculture, forestry, animal husbandry and fishery	256.19	259.74
CAP	Human capital: expressed by the average years of education of rural residents	7.34	1.18
ECO	Regional economic development level: expressed in regional per capita GDP	32.21	25.43
RES	Resource endowment: expressed in rural per capita agricultural machinery value	7.41	1.10

## Empirical results and analysis

### Panel regression test

**Table 3** reports the empirical effects of environmental regulation on the rationalisation and optimisation of agricultural industrial structure. It is clear that whether it is random effect regression or fixed effect regression, environmental regulation has a significant negative impact on rationalisation and a significant positive impact on optimisation of agricultural industrial structure.(As mentioned above, the Thiel Index is negatively correlated with the rationalization of agricultural industrial structure, and the agricultural carbon emission is negatively correlated with the intensity of environmental regulations. Therefore, the regression result means that environmental regulation has a negative impact on the rationalization of agricultural industrial structure and a positive impact on the upgrading of agricultural industrial structure.) These findings are consistent with the theoretical analysis presented in the current study, and previous reports. A multicollinearity test is performed to verify the validity of the regression results, with the result showing that the variance inflation factor (VIF) is less than 10, indicating no substantial multicollinearity problem between variables. A Housman test was also carried out, showing that the null hypothesis is rejected at the 1% significance level and that fixed-effects regression was better than random-effects regression. Given this and based on the panel fixed-effect regression results, the coefficient of environmental regulation on the rationalisation of agricultural industrial structure is -0.145, illustrating a 1% significance level test. The coefficient of the optimisation of agricultural industrial structure is 0.019, also fulfilling the 1% significance level test. Consequently, a 0.145% decline in rationalisation of agricultural industrial structure and a 0.019% increase in optimisation of agricultural industrial structure are related to each percentage point increase in environmental regulation intensity, validating hypotheses 1 and 2.

**Table 3:** Panel regression results.

Variable	Rationalisation of agricultural industrial structure		Optimisation of agricultural industrial structure	
	RE	FE	RE	FE
ER	-0.109***	-0.145***	0.008***	0.019***
	(0.027)	(0.029)	(0.003)	(0.004)
URB	0.199***	0.227***	0.004	0.009
	(0.055)	(0.054)	(0.007)	(0.008)
SUP	0.0002***	0.0002***	8.91e-06**	6.01e-06
	(3.01e-05)	(2.95e-05)	(4.54e-06)	(4.61e-06)
CAP	0.001	1.05e-05	0.001	0.0004
	(0.009)	(0.009)	(0.001)	(0.001)
ECO	0.0001**	0.001*	1.88e-06	-6.31e-05
	(0.0004)	(0.0004)	(6.11e-05)	(6.86e-05)
RES	-0.037*	-0.037*	0.007**	0.009***
	(0.020)	(0.020)	(0.003)	(0.003)
Constant	2.047***	2.200***	-0.015	0.029
	(0.146)	(0.144)	(0.016)	(0.022)
Time dummy variable	control	control	control	control
Observations	589	589	589	589
F/LR Chi2 /Wald Chi2	258.05***	12.42***	1414.27***	30.07***

Note: T values are in parentheses, *, **, and ***, representing significance at 10%, 5%, and 1% levels, respectively. Same below.

Regarding the control variables, URB, SUP, and ECO significantly positively impact the rationalisation of agricultural industrial structure, where the larger the control variable, the worse the rationalisation. This outcome is consistent with studies by Qin Dezhi [48]. However, RES significantly negatively impacts the rationalisation of agricultural industrial structure. It may be because the increase in agricultural mechanisation investment directly improves agricultural production conditions, promotes agricultural labour and production efficiency, and makes the input and output of agricultural industries more balanced. At the same time, RES has a significant positive impact on optimising the industrial structure because agricultural mechanisation is one of the main directions for optimising and upgrading agricultural production factors, and the level of agricultural machinery reflects the modernisation of factors to a certain extent. However, the input of advanced production factors will inevitably induce the advanced evolution of agricultural industrial structure with high added value and modern industrial form.

### Heterogeneity test

The impact of environmental regulation on agricultural industrial structure is not necessarily universal. Therefore, it is necessary to use sub-samples to test the heterogeneity. This paper continued to explore the heterogeneous impact of environmental regulation from the two aspects of structural differences and regional differences.

#### (1) Structural heterogeneity

Distinct from linear regression models, quantile regression can be used to estimate the heterogeneous effects of independent variables on dependent variables at different quantile points, which can better describe the global characteristics of the conditional distribution of dependent variables. Moreover, it can relax the influence of strict error terms and outliers and has high robustness. Using the panel quantile regression method, The current study conducted a quantitative test on Hypotheses (2) and (3) to analyse the industrial adjustment effects of environmental regulation at different levels of industrial structure. Considering sample size characteristics and sample standard deviation, three agricultural industrial structure level quantiles of 0.25, 0.5, and 0.75 were selected. These represented the low, medium, and high agricultural industrial structure levels.

Data in **Table 4** reports the panel quantile regression results of environmental regulation on agricultural industrial structure adjustment. The impact of environmental regulation on rationalisation is consistently negative. However, the regression results on the 25% quantile are insignificant, and the regression coefficients on the 50% and 75% quantiles are significant at the 5% level. Considering that the smaller the Taylor index is, the higher the rationalisation level of an agricultural industrial structure, quantile regression shows that the rationalisation adjustment effect of environmental regulation on an agricultural industrial structure is larger at the middle and low rationalisation level of agricultural industrial structure, larger than the samples at the high rationalisation level of agricultural industrial structure. The impact of environmental regulation on the optimisation of agricultural industrial structure is uniformly positive. However, only the regression results in the 25% and 50% quantiles are significant at the 5% significance level, with the regression results at the 75% quantiles are insignificant. Such outcomes indicate that environmental regulation has a more significant influence on the adjustment of the high-grade samples of the middle and low agricultural industrial structure than that of the high-grade samples of the high agricultural industrial structure. With a continuous improvement of the level of agricultural industrial structure, the impact of environmental regulation on the adjustment of the industrial structure has gradually weakened. Alternatively, when the level of rationalisation and optimisation of the agricultural industrial structure reaches a high level, the space and extent of the adjustment are ’saturated’. On the other hand, high-level agricultural industrial structure may have completed the stage task of ’regulatory adjustment’ and already possess the elements and industrial connotation of high-quality development. Therefore, the environmental regulation’s industrial structure adjustment effect will no longer function.

**Table 4:** Quantile regression results.

Variable	Rationalisation of agricultural industrial structure			Optimisation of agricultural industrial structure		
	Q25	Q50	Q75	Q25	Q50	Q75
ER	-0.154	-0.145**	-0.136**	0.018**	0.019**	0.020
	(0.115)	(0.064)	(0.060)	(0.008)	(0.009)	(0.016)
URB	0.276	0.225*	0.178	0.016	0.010	0.002
	(0.243)	(0.136)	(0.128)	(0.013)	(0.015)	(0.027)
SUP	0.002	0.0001**	0.0002**	3.68e-06	5.67e-06	8.14e-06
	(0.001)	(0.001)	(7.61e-05)	(8.85e-06)	(9.60e-06)	(1.80e-05)
CAP	-0.001	0.0004	0.001	-0.001	0.0002	0.001
	(0.035)	(0.020)	(0.019)	(0.004)	(0.004)	(0.007)
ECO	0.001	0.001	0.001	-0.0001	-0.0001	-2.50e-05
	(0.002)	(0.001)	(0.001)	(0.0001)	(0.0001)	(0.0003)
RES	-0.055	-0.036	-0.019	0.007	0.009	0.0112
	(0.080)	(0.045)	(0.042)	(0.006)	(0.0070)	(0.013)
Time virtual variable	control	control	control	control	control	control
Observations	589	589	589	589	589	589

#### (2) Regional heterogeneity

China is a vast country with noticeable differences in economic and social conditions across different provinces or regions. Different results may appear in sub-regional studies of the same problem, which can better reveal its practical logic. The current study divided the 31 sample provinces into eastern, central, and western regions according to geographical location and continued to use the panel fixed-effect model for regression testing (**Table 5**).

**Table 5:** Sub-regional panel regression results.

Variable	Rationalisation of agricultural industrial structure			Optimisation of agricultural industrial structure		
	East	Central	West	East	Central	West
ER	0.083	-0.252 ***	-0.138 **	0.015**	0.059***	-0.011
	(0.064)	(0.039)	(0.063)	(0.007)	(0.009)	(0.011)
URB	0.149	0.075	0.226 *	0.052***	-0.024*	0.011
	(0.154)	(0.053)	(0.131)	(0.017)	(0.012)	(0.023)
SUP	0.0003 ***	6.55e-05	0.0001 *	1.14e-05**	5.30e-08	-6.77e-07
	(4.69e-05)	(5.71e-05)	(5.82e-05)	(5.22e-06)	(1.31e-05)	(1.02e-05)
CAP	0.014	0.004	0.017	-0.006**	0.009***	-0.005*
	(0.025)	(0.010)	(0.016)	(0.003)	(0.002)	(0.003)
ECO	3.60e-05	-0.001	-0.001	-0.0002**	0.0004**	0.0002
	(0.001)	(0.001)	(0.002)	(8.03e-05)	(0.0002)	(0.0003)
RES	-0.063	0.043**	-0.101 **	0.006	0.012***	0.011
	(0.045)	(0.018)	(0.044)	(0.005)	(0.004)	(0.008)
Constant	1.077 ***	2.382 ***	2.499 ***	0.049	0.179***	-0.090
	(0.287)	(0.261)	(0.359)	(0.032)	(0.060)	(0.063)
Time virtual variable	control	control	control	control	control	control
Observations	209	190	190	209	190	190
F/LR Chi2 /Wald Chi2	204.14 ***	674.51 ***	207.51 ***	16.98 ***	16.52 ***	45.83 ***

First, as far as the central and western regions are concerned, the impact of environmental regulation on the rationalisation of the agriculture industry is significantly negative at the 5% level. However, the coefficient of environmental regulation in the eastern region is positive but insignificant. In other words, environmental regulation inhibits the rationalisation of agricultural industrial structure in the central and western provinces but has no significant effect on the eastern provinces. Perhaps, compared with the central and western regions, there is increased structure level and complexity in the eastern region, the agricultural industry is also fully integrated with the secondary and tertiary industries, and the industry has reached the stage of collaborative internal and external development. The early implementation of environmental regulation in the eastern region will also result in more vigorous regulation intensity and policy implementation. Environmental regulation mainly exhibits the effect of ’innovation compensation’, which is less affected by the combined structure of industries and more manifested in upgrading factors and industries.

Second, in the eastern and central regions, the impact of environmental regulation on the optimisation of the agricultural industrial structure is significantly positive at the 5% level, but is negative and insignificant in the western region. In other words, environmental regulation can promote the high-grade adjustment of agricultural industrial structure in the eastern and central provinces, but has no apparent impact on the western provinces. Compared with the eastern region, the agricultural production mode in the western region is traditional, and the level of structure is relatively low. The ’compliance cost’ effect mainly occurs under the exogenous impact of environmental regulation. The western region with weak regulation have become the elements with high regulation costs and the objects of regional transfer of industrial sectors. The elements and industrial upgrading have not yet occurred.

### Panel threshold regression test

With the change in regulation intensity, will the impact of environmental regulation on the adjustment of agricultural industrial structure remain unchanged? In other words, will there be a non-linear relationship or threshold effect between environmental regulation and agricultural industrial structure? Based on the above considerations, this paper used the panel threshold regression model to empirically test (4) and (5) to explore the threshold effect of environmental regulation.

The threshold effect test and the threshold estimation value test were carried out, as shown in **Table 6**, using environmental regulation as the threshold variable. The simulation of the likelihood ratio was repeated 300 times by the Bootstrap method. The test results show that the F statistic of the single threshold in the model (4) is significant at the 5% statistical level, and the double and triple threshold tests do not pass the significance test. The single, double, and triple threshold tests (5) fail the significance test. Therefore, there is a single threshold non-linear relationship between environmental regulation and the rationalisation of agricultural industrial structure. However, there is no non-linear relationship of threshold effect between environmental regulation and the optimisation of agricultural industrial structure.

**Table 6:** Threshold effect test results.

Variable	Threshold type	F value	P value	Critical value		
				10%	5%	1%
Rationalisation of agricultural industrial structure	Single threshold test	67.62	0.04	56.073	63.8029	89.3168
	Double threshold test	31.59	0.52	98.6750	122.4818	156.9102
	Triple threshold test	34.92	0.33	60.9549	72.5358	105.1296
Optimisation of agricultural industrial structure	Single threshold test	29.08	0.24	37.6774	41.0485	54.9859
	Double threshold test	1.20	1.00	33.1855	37.2721	50.8189
	Triple threshold test	10.43	0.86	31.5276	38.3672	54.3165

**Table 7** shows the full-sample and sub-regional panel threshold regression results for environmental regulation and agricultural industrial structure rationalisation. (1) The environmental regulation level restricts environmental regulation’s influence on rationalisation and presents non-linear characteristics. Specifically, when the regulation intensity is less than the threshold value of 4.1563, the elastic coefficient of the rationalisation adjustment effect of the agricultural structure of environmental regulation is -0.211; when it is greater than the threshold value of 4.1563, the elastic coefficient is -0.154, and all passed the 1% significance level test. It can be seen that with the increase in the intensity of environmental regulation, its negative impact on the rationalisation of agricultural industrial structure tends to weaken. After crossing the threshold, the impact intensity of environmental regulation decreases by nearly six percentage points, demonstrating that the current impact of environmental regulation on the agricultural industrial structure rationalisation is still in the ’following costs’ stage. However, the strengthening of regulation reduces its impact, indicating that the allocation of resources, factors and industries has gradually changed from mindlessly reducing the cost of regulation to an orderly and reasonable adjustment. It is conceivable that with the continuous implementation and strengthening of environmental regulation policies, its negative impact on the rationalisation of agricultural industrial structure will continue to weaken, and even a positive transformation will occur, becoming a ’key force’ in promoting the rational adjustment of agricultural industrial structure.

**Table 7:** Panel threshold regression results.

Variable	Rationalisation of agricultural industrial structure			
	Full sample	East Region	Central Region	Western Region
ER	-	-	-	-
	-	-	-	-
ER< = φ	-0.211***	0.115	-0.207***	-0.029
	(0.029)	(0.059)	(0.039)	(0.058)
ER> φ	-0.154***	0.148	-0.198***	-0.093*
	(0.027)	(0.058)	(0.040)	(0.056)
URB	0.152***	0.109	0.0595	0.363***
	(0.052)	(0.139)	(0.051)	(0.118)
SUP	0.0002***	0.0002***	4.25e-05	0.0001**
	(2.83e-05)	(4.29e-05)	(5.46e-05)	(5.14e-05)
CAP	-0.001	0.008	0.005	0.020
	(0.009)	(0.023)	(0.009)	(0.014)
ECO	0.001*	0.0002	-0.0006	-0.0006
	(0.0004)	(0.001)	(0.001)	(0.002)
RES	-0.0348*	-0.0778*	0.0364**	-0.148***
	(0.019)	(0.041)	(0.018)	(0.040)
Constant	2.306***	1.060***	2.193***	2.509***
	(0.138)	(0.258)	(0.252)	(0.317)
Time virtual variable	control	control	control	control
Observations	589	209	190	190
F test	325.97***	179.05***	739.8***	269.01***

(2) There is no significant threshold effect in the eastern region, and the regression results are positive but insignificant. There is a single threshold effect in the central region. The coefficients of environmental regulation before and after the threshold value are negative and pass the 1% significance test. The coefficient of environmental regulation before and after the threshold value is negative, passing the 1% significance test. The regression results show that after the intensity of environmental regulation exceeds the threshold value of 6.03, the impact of its agricultural structure rationalisation decreases from -0.207 to -0.198, showing a slight decrease. There is a single threshold effect in the western region. However, the environmental regulation coefficients significantly differ before and after the threshold value. When the environmental regulation intensity is lower than the threshold value of 3.5522, the rationalisation adjustment effect of environmental regulation on the agricultural structure is negative but insignificant. Only when the environmental regulation intensity is higher than the threshold value does the environmental regulation have a negative effect. That is to say, to exert the industrial structure adjustment effect of environmental regulations in the Western region, a relatively high degree of regulation policy and enforcement intensity is required.

## Robustness test

### Replacing explanatory variables

The agricultural carbon emission index is used to calculate environmental regulation intensity, and the results are generally in line with the theoretical expectations of this study. Consequently, this study will further test the robustness of the regression results by replacing variables. According to the data of China’s State Grain Administration, the cost of fertiliser accounts for the most substantial proportion of the average cost of grain in China, accounting for about 30% of the total cost. At the same time, fertiliser abuse is agriculture’s most crucial pollution source. Therefore, one of the main contents of environmental regulation policy is to reduce the use of chemical fertilisers. In previous studies [49], the pollution source was used as the proxy variable of environmental regulation, and the amount of fertiliser was used to represent the intensity of environmental regulation. An empirical test was applied to the data, and regression analysis data are shown in **Table 8**. The outcomes are (1) The panel fixed effect regression test shows that environmental regulation negatively impacts the rationalisation of agricultural industrial structure and positively impacts the optimisation of agricultural industrial structure, consistent with results detailed previously, and (2) A significant threshold effect between environmental regulation and the rationalisation of agricultural industrial structure is depicted. The regression coefficient decreases significantly after the threshold value, consistent with the existing regression results.

**Table 8:** Regression results with replaced explanatory variables.

Variable	Panel instrumental variable regression		Panel threshold model
	Rationalisation of agricultural industrial structure	Optimisation of agricultural industrial structure	Rationalisation of agricultural industrial structure
ER	-0.1315***	0.0030*	-
	(0.0491)	(0.0067)	-
ER< = φ	-	-	-0.165***
			(0.0251)
ER >φ	-	-	-0.131***
			(0.0240)
URB	0.2267**	0.0085	0.187***
	(0.1197)	(0.0127)	(0.0531)
POL	0.0002**	9.67e-06	0.0002***
	(2.85e-05)	(4.11e-06)	(2.79e-05)
EDU	-0.0017	-0.0002	-0.003
	(0.0092)	(0.0038)	(0.0088)
GDP	0.0009	-0.00003	0.0010**
	(0.0008)	(0.0001)	(0.0004)
RES	-0.0450	0.0061	-0.0425**
	(0.0382)	(0.0058)	(0.0181)
Constant	2.1472***	0.0131	2.169***
	(0.2887)	(0.0362)	(0.1363)
Time virtual variable	control	control	control
Observations	589	589	589
F test	11.84***	31.63***	322.49***

### Robustness tests considering endogeneity problems

Theoretically, the introduction of environmental regulation policies and the degree of regulation is mainly affected by the national agricultural development strategy—not endogenous to agricultural industrial structure—and can be regarded as strict exogenous variables. However, the strategic appeal for high-quality agricultural development includes optimising or improving the agricultural industrial structure and the basic connotation of high-quality agricultural environmental development. Therefore, there may be endogenous problems because of the reciprocal causation between the adjustment of agricultural industrial structure and environmental regulation. Referring to the endogeneity treatment method of Han et al. [50], this paper used the first-order lagged term of environmental regulation as an instrumental variable to conduct panel regression again as a robust regression analysis (**Table 9**).

**Table 9:** Instrumental variable regression results.

Variable	Panel instrumental variable regression		Panel threshold model
	Rationalisation of agricultural industrial structure	Optimisation of agricultural industrial structure	Rationalisation of agricultural industrial structure
ER	-0.147***	-0.022***	-
	(0.031)	(0.005)	-
ER< = φ	-	-	-0.191***
			(0.028)
RR> φ	-	-	-0.146***
			(0.027)
URB	0.195***	0.008	0.139***
	(0.055)	(0.009)	(0.053)
SUP	0.0002***	4.19e-06	0.0002***
	(2.85e-05)	(4.77e-06)	(2.79e-05)
CAP	0.002	-0.0003	-0.001
	(0.009)	(0.002)	(0.009)
ECO	0.001**	-4.38e-05	0.001**
	(0.0004)	(7.17e-05)	(0.0004)
RES	-0.0354*	0.0112***	-0.040**
	(0.020)	(0.003)	0.139***
Constant	2.204***	0.031	2.225***
	(0.147)	(0.025)	(0.175)
Time dummy variable	control	control	control
Observations	558	558	558
F test	385.78***	35.05***	354.29***

The outcomes are as follows: (1) The two-stage least squares method (2SLS-IV) was adopted for the regression test, and the results are shown in Table 9. Considering endogeneity, environmental regulation has negative and positive effects on the rationalisation and optimisation of agricultural industrial structure, and both are significant at the 1% significance level. The estimated coefficient is not much different from the above, which conforms to the theoretical and empirical results, (2) To study the threshold effect, the paper estimated the panel threshold of the model again by lagging the environmental regulation indicators by one period. Table 9 shows that the threshold effect of environmental regulation on the rationalisation of agricultural industrial structure is still significantly negatively correlated, with weakening characteristics, which have not changed significantly compared with the previous estimation results, and (3) Compared with the panel threshold regression results above, the environmental regulation coefficient lagging by one period is significantly smaller, which shows that the impact of environmental regulation on the rationalisation of agricultural industrial structure is continuous. Early environmental regulation policies also specifically affect the adjustment of agricultural industrial structure. However, the impact of environmental regulation in the current period is more significant; this may be because the environmental regulation policies in the current period tend to be better and enforced more strongly than in the previous period.

## Conclusions and discussion

Unlike previous reports, which focused on investigating the impact of environmental regulations on the structural adjustment of the primary, secondary, and tertiary industries, this study focuses on the agricultural industry. It analyses the structural adjustment issues of environmental regulations on different industrial sectors within the agricultural industry, which has certain novelty in the topic selection. Meanwhile, based on the long panel data of 31 Chinese provinces between 2000 and 2018, a combination of panel regression, panel threshold regression, and instrumental variable regression models for empirical testing was undertaken, producing results with high credibility and robustness.

These data also explain two key questions. First, at the theoretical level, does the ’pollution paradise effect’ or the ’Porter effect’ exist when adjusting agricultural industrial structure? The conclusion proves: (1) There is a ’compliance cost effect’ between environmental regulation and the rationalisation of agricultural industrial structure: every 1% increase in environmental regulation will reduce the rationalisation of agricultural industrial structure by 14.5%, (2) There is an ’innovation compensation effect’ between environmental regulation and the optimisation of agricultural industrial structure: every 1% increase in environmental regulation will increase the optimisation of agricultural industrial structure by 1.9%, and (3) Moreover, as the intensity of regulation changes, there is a non-linear relationship between environmental regulation and the rationalisation of agricultural industrial structure with threshold characteristics. At the same time, there is only a linear relationship with the optimisation of agricultural industrial structure.

Second, at the practical level, whether the unbalanced and uneven characteristics of regional development in China’s vast territory will be mapped to the adjustment of agricultural industrial structure. The heterogeneity test found that environmental regulation significantly impacts the rationalisation and optimisation of agricultural industrial structure at the low and medium levels of agricultural industrial structure. Environmental regulation is not conducive to rationalising agricultural industrial structure in the central and western regions. However, it is instrumental in optimising agricultural industrial structure in the eastern and central regions. Moreover, there is a significant threshold non-linear relationship in central and western regions, while the threshold characteristics in the eastern region are not significant.

Based on the above conclusions, the following policy recommendations are suggested: On the one hand, the Chinese government should continue to adhere to the path of environmental regulation and promote the improvement and intensity of the environmental regulation system. After passing the "painful period" of ’compliance cost effect’, the agricultural industry structure will naturally develop in the direction of rationalisation and optimisation. Alternatively, environmental regulation policies should be targeted and inclusive. In particular, local governments should formulate and introduce environmental regulation policies that align with the level of local economic and social development and the status of agricultural industry structure to effectively adjust agricultural industrial structure.

## Limitations and future research

The research outlined in this report has certain limitations. Although the impact of environmental regulation on the adjustment of agricultural industrial structure is investigated, a mechanism test is absent, not revealing the factors through which environmental regulation will affect the adjustment of agricultural industrial structure. Differentiated impacts of different types of environmental regulations on the adjustment of agricultural industrial structure are also absent. Therefore, it is necessary for future studies to distinguish between formal environmental regulation and informal environmental regulation, and to deeply explore the mediating effect or moderating effect of variables such as farmers’ cognition, power allocation and technological innovation, to empirically test the internal mechanism of environmental regulation on agricultural industrial structure adjustment. Finally, with more data available, a questionnaire could be used to explore the impact of environmental regulation on farmers’ planting structure adjustments.

## Supporting information

S1 Data (XLS)
